# Image Quality Standardization in Radiomics: A Systematic Review of Artifacts, Variability, and Feature Stability

**DOI:** 10.3390/s26031039

**Published:** 2026-02-05

**Authors:** Francesco Felicetti, Francesco Lamonaca, Domenico Luca Carnì, Sandra Costanzo

**Affiliations:** 1 Department of Computer Engineering, Modeling, Electronics and Systems Engineering, University of Calabria, Ponte P. Bucci, 87036 Rende, Italy; 2 Institute of Nanotechnology (CNR-NANOTEC), National Research Council of Italy, 87036 Rende, Italy; 3 Consorzio Nazionale Interuniversitario per le Telecomunicazioni, 43124 Parma, Italy; 4 Inter-University National Research Center on Interactions Between Electromagnetic Fields and Biosystems (ICEmB), 16126 Genova, Italy

**Keywords:** image quality assessment, measurements, artifacts, radiomics, feature extraction, standardization, personalized medicine

## Abstract

This paper explores the role of metrology in the assessment of image quality in the field of radiomics. Image Quality Assessment (IQA) is central to ensuring the reliability and reproducibility of radiomic analyses, as it directly affects the accuracy of feature extraction and segmentation, ultimately impacting diagnostic outcomes. From the analysis of approximately 20,000 papers sourced from three databases (PubMed, Scopus, IEEE Xplore), last searched in December 2025, the need for standardized imaging protocols and quality control measures emerges as a critical theme. Studies were included if they involved radiomic feature extraction and evaluated the impact of image quality variations on feature robustness and no formal risk-of-bias assessment was performed. A total of 105 studies were included, covering different medical imaging modalities. Across the included studies, noise, motion, acquisition and reconstruction parameters, and other artifacts consistently emerged as major sources of radiomic feature instability. Indeed, in most papers, IQA is neglected, while the effect of poor-quality images is reported. This research identifies and discusses the relevant issues reported in clinical practice, as well as the main metrics adopted for image quality evaluation. Through a comprehensive review of current literature and an analysis of emerging trends, this paper highlights the urgent need for innovative solutions in image quality metrics tailored to radiomics applications.

## 1. Introduction

Radiomics is revolutionizing personalized medicine and oncology through its potential to enhance diagnosis, predict prognosis, and improve therapeutic responses from non-invasive imaging [1,2,3,4,5]. The core of this discipline relies on high-quality imaging data, as the accuracy of radiomic analysis is strictly related to the quality of the input images [6]. IQA is thus essential in ensuring the reliability and reproducibility of radiomics applications. Image Quality Assessment (IQA) covers the evaluation of various aspects of the image, such as resolution, contrast, noise, and artifacts, which can significantly impact the reliability of feature extraction and subsequent analysis [7,8,9,10,11]. First, it acts as a quality control filter, accurately screening images and excluding those that fail to meet minimum quality benchmarks. Second, it ensures that the data entering the radiomics pipeline are of sufficient quality to yield meaningful insights [12]. This is a crucial step because the robustness of radiomic features is highly dependent on the consistency and quality of the imaging data [13,14]. Variations in imaging protocols, scanner types, and even patient positioning can introduce biases, thus affecting the reproducibility of the results [15,16,17]. Moreover, advanced IQA methods can play a major role in standardizing images across different institutions and scanner types, facilitating multicentric studies and the generalization of radiomics models [18]. The introduction of automated tools for image assessment is becoming increasingly urgent because an efficient IQA tool could rapidly evaluate vast datasets [19,20], identifying, measuring, and, when possible, correcting image quality issues. This would support manual operators by reducing their workload and eliminating elements related to the subjectivity of a visual inspection [21]. In summary, the importance of IQA in radiomics applications cannot be overstated. It is an essential prerequisite for the accurate extraction and analysis of radiomic features, directly influencing the predictive power and clinical utility of radiomics models. As the field advances, ongoing developments in IQA methodologies will be critical to harnessing the full potential of radiomics in personalized medicine and beyond. When addressing image quality challenges in radiomics, it is important to distinguish between two complementary approaches: image quality standardization and image quality correction. Standardization refers to protocol-level control measures implemented at the acquisition stage to ensure consistent imaging parameters across scanners, institutions, and time points [18,22,23]. This includes harmonized acquisition protocols, calibration procedures, and quality control measures designed to prevent quality degradation before it occurs. In contrast, correction refers to algorithmic, post-acquisition interventions applied to mitigate degradations already present in acquired images, such as denoising algorithms [24,25,26], artifact reduction techniques [26,27], and harmonization methods [28,29,30]. While both approaches are essential and complementary, standardization should be prioritized, as it reduces variability at the source, whereas post hoc corrections may introduce additional uncertainty or alter radiomic feature distributions unpredictably. This paper aims to critically examine the role of IQA in radiomics by analyzing its presence and consideration in the existing scientific literature, trying to identify the IQA impact on radiomic feature extraction and diagnostic reliability.

While previous systematic reviews have addressed radiomic feature reproducibility or harmonization strategies, this review systematically quantifies the prevalence of IQA consideration in radiomics literature from a metrological perspective. Unlike existing reviews [14,18,28,29,30], primarily focusing on clinical or algorithmic aspects, our work introduces the following new aspects: (1) a quantitative analysis of the IQA gap in radiomics research, (2) a first attempt at systematic categorization of artifacts linked to affected feature classes, (3) a critical comparison of correction approaches, (4) practical recommendations for integrating IQA as a quality-control step in radiomics pipelines.

## 2. Materials and Methods

This systematic review was conducted following the PRISMA 2020 statement [31], with the primary objective of mapping and analyzing how image quality affects radiomic feature extraction and the stability of downstream predictive models. The focus of this review is methodological rather than clinical, aiming to provide a comprehensive overview of experimental designs, quality metrics, and correction strategies employed in radiomics research to mitigate the impact of image degradation. The literature search was carried out in three major scientific databases (PubMed, Scopus, and IEEE Xplore) to comprehensively capture biomedical, engineering, and computational imaging studies relevant to radiomics and IQA. Searches were conducted in each database up to December 2025, with no language or publication-type restrictions. An initial broad exploratory query using the single keyword “Radiomics” retrieved approximately 20,000 records across sources. To focus on works explicitly addressing IQ-related degradations in the context of radiomics, we then applied a refined query that combined Radiomics with IQA terms (noise, blur, artifact, image perturbation, image quality metrics). The exact database-specific queries were:

- **Scopus:**
  TITLE-ABS-KEY (radiomics) AND (TITLE-ABS-KEY (noise)
  OR TITLE-ABS-KEY (artifact)
  OR TITLE-ABS-KEY (blur) OR TITLE-ABS-KEY (“image perturbation”)
  OR TITLE-ABS-KEY (“image quality metrics”))
- **PubMed:**
  (Radiomics) AND ((Noise) OR (Blur) OR (artifact)
  OR (“image perturbation”) OR (“image quality metrics”))
- **IEEE Xplore:**
  ((“Document Title”:“radiomics”) OR (“Abstract”:“radiomics”))
  AND (“noise” OR “blur” OR “artifact” OR “image perturbation”
  OR “image quality metrics”)

All search results were exported and merged into a unified dataset. Duplicates were identified and removed using semi-automated filtering followed by manual verification. The combined corpus served as the foundation for the subsequent screening and eligibility assessment.

Inclusion and exclusion criteria were defined *a priori*, according to the PICO framework adapted for methodological systematic reviews:

- **Population:** Imaging studies (CT, MRI, PET, or hybrid modalities) employing radiomic feature extraction.
- **Intervention/Exposure:** Image quality variations or artifacts, whether simulated, measured, or induced experimentally (e.g., noise, blur, motion, beam hardening, or acquisition parameters).
- **Comparator:** Reference or baseline imaging conditions, or different levels of degradation.
- **Outcome:** Quantitative assessment of feature variability, robustness, or reproducibility in relation to IQA metrics.

**Figure 1:**
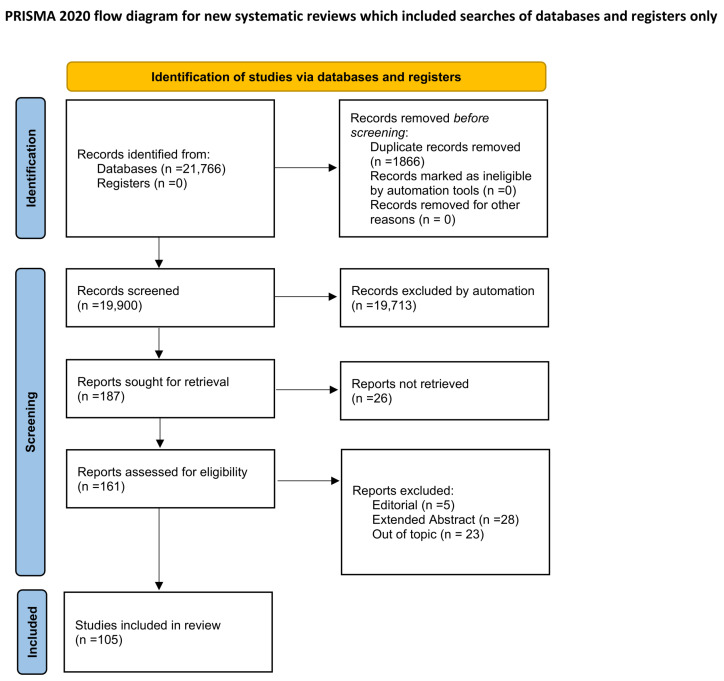
PRISMA 2020 flow diagram illustrating the study identification, screening, and inclusion process. A total of 21,766 records were retrieved from databases (PubMed, Scopus, IEEE Xplore), and 1866 duplicates were removed prior to screening. After title and abstract screening (19,990 records), 187 reports were sought for full-text retrieval, of which 26 could not be accessed. Among the 161 reports assessed for eligibility, 59 were excluded for the following reasons: editorial (*n* = 5), extended abstract (*n* = 28), and out of topic (*n* = 23). Finally, 105 studies were included in the qualitative synthesis [31].

Studies were included if they met all the above conditions. Works that were reviews, editorials, or methodological protocols without original data, as well as those focusing exclusively on image quality without radiomic analysis, were excluded. A total of 21,766 records were retrieved, and 1866 duplicates were removed, leaving 19,990 unique records for title and abstract screening. After preliminary filtering, 187 reports were sought for full-text assessment, of which 26 could not be retrieved. The remaining 161 reports were evaluated for eligibility, and 23 were excluded for specific reasons: reviews or protocols (*n* = 10), radiomics without quantitative IQA metrics (*n* = 7), IQA without radiomics (*n* = 2), insufficient data (*n* = 3), and duplicate datasets (*n* = 1). At this stage, other 33 studied were were excluded for the following reasons: Extended abstract (*n* = 28), Editorial (*n* = 5). Consequently, 105 studies were included in the final qualitative synthesis. The overall study selection process is summarized in Figure 1, according to the PRISMA 2020 flow diagram.

## 3. Methodological and Scope Limitations

Since this systematic review was conducted with specific methodological adaptations justified by its exploratory and metrological scope, it was not prospectively registered (e.g., in PROSPERO [32]), and no formal risk-of-bias assessment, reporting-bias assessment, or certainty-of-evidence evaluation (e.g., QUADAS-2, GRADE [33,34]) was performed. These procedures are designed for clinical intervention or diagnostic accuracy studies, so they are not applicable to a methodological review that does not focus on quantitative estimations or clinical outcomes. However, it uniquely addresses image quality considerations in radiomics research. No inferential statistical analyses or meta-analytic pooling were performed due to substantial heterogeneity in imaging modalities (CT, MRI, PET, ultrasound), outcome metrics (ICC, SNR, CNR, Dice coefficients), and experimental designs (phantom vs. clinical studies). Data were instead qualitatively analyzed by emphasizing trends, methodological gaps, and reproducibility issues identified across the retrieved literature.

It is important to further clarify that the specific choice of the search queries adopted in the present review may have introduced selection bias. As stated in the Introduction, the adopted strategy was intentionally designed to focus on studies explicitly addressing image quality degradation and artifacts in radiomics through specific terminology (noise, artifact, blur, image perturbation, image quality metrics). Consequently, works indirectly dealing with image quality, such as studies primarily focused on preprocessing, harmonization strategies, robustness analyses, or downstream pipeline optimization, may not have been captured if image quality issues were not explicitly mentioned in the title, abstract, or keywords.

Furthermore, the bibliometric analysis was based on three major scientific databases (PubMed, Scopus, IEEE Xplore), providing comprehensive coverage of biomedical and engineering literature. Although this choice ensures broad coverage of peer-reviewed biomedical and engineering research, it may have excluded some studies, potentially limiting the full completeness of the bibliometric analysis.

## 4. Radiomics and Image Quality

**Figure 2:**
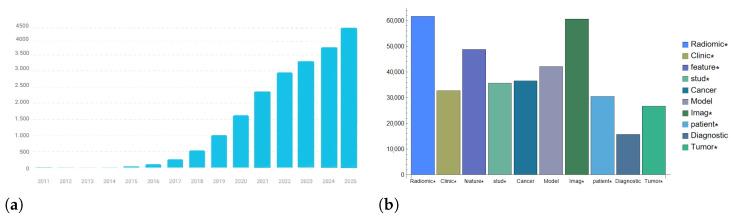
(**a**) Distribution of scientific articles containing the word “radiomics” divided by year of publication [36]. (**b**) Histogram of the most frequent words in radiomics literature. The asterisk groups words with the same root (e.g., Imag* includes image, images, imaging).

Searching for the word “radiomics” across various scientific databases yields a considerable number of results. Specifically, a search for “radiomics” in titles, abstracts, and keywords on Scopus, PubMed, and IEEE Xplore databases finds just under 20,000 unique results. The topic is of clear scientific interest, as evidenced by the increasing number of studies on the topic (Figure 2a). The analysis of the most frequent words in this research field reveals “radiomics” as the main topic, but other prominent words include “feature” or “features” and “image/images/imaging” (Figure 2b). The term “radiomics” was introduced by Lambin et al. in 2012 [35], marking its first appearance in the scientific literature to describe the extraction of quantitative features from radiological images for cancer studies using computed tomography (CT). This introduction discusses the use of the term “radiomics” in the context of medical imaging and analysis, focusing on the extraction of numerous quantitative features from medical images for various clinical applications, including but not limited to cancer research. Although the attribution of this term to the mentioned study is clear, it is also necessary to state that previous studies exist that discuss the extraction of information from medical images [7,8,9].

From the analysis of the most frequent words and the definition of radiomics, it becomes clear that images play a central role in the radiomic workflow. Images can indeed be considered the fundamental link connecting data acquisition to the extraction of information, which allows qualitative or quantitative evaluations and provides the basis for constructing prognostic models. In a more detailed examination of the same databases previously considered, by performing refined queries to include not just “Radiomics” but also one of the following terms: “noise”, “artifact”, “blur”, “image perturbation,” or “image quality metrics” (each highlighting the crucial role of image quality), the initial set of nearly 20,000 results reduced to less than 750 works. This reduction to 3.75% is clearly in contrast with expectations, especially considering the absence of an established and acknowledged effective metric for image evaluation. The question that soon arises is whether it is appropriate to introduce an analysis of image quality within the radiomic workflow. The location and selection activity was carried out with the help of the MySLR digital platform [36].

## 5. Image Quality Assessment Tools: A Need

**Figure 3:**
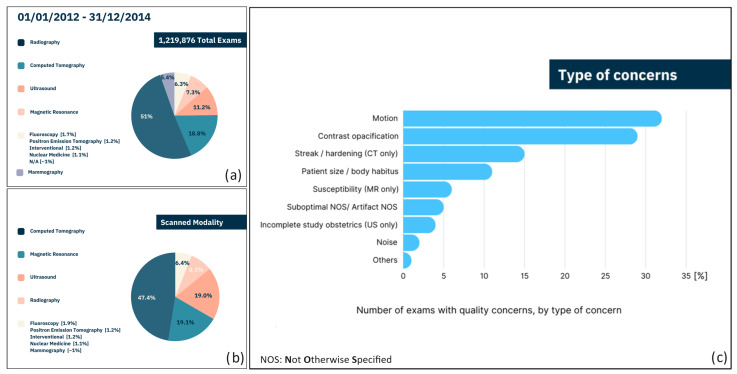
Infographics related to medical imaging over a three-year period from 1 January 2012, to 31 December 2014, according to [37]. (**a**) Pie chart representing the distribution of 1,219,876 total exams across different scanning modalities. (**b**) Pie chart representing a subset of the data from panel (**a**), showing the distribution of exams for which an imaging defect was identified. (**c**) Bar chart detailing the number of exams with quality concerns, by type of concern. The concerns are quantified as percentages of the total number of quality concerns observed.

Altered images in radiology can significantly impact patient care, leading to diagnostic inaccuracies that may result in misdiagnosis or delayed treatments. This not only increases patient’s anxiety and dissatisfaction but also increases the risk of legal consequences. According to a 2018 study [37] conducted on a sample of 1,210,858 radiology exams of various types (Figure 3), concerns about image quality were reported in 2.4% of cases during clinical evaluation. Although this percentage may seem almost negligible, it is crucial to consider several aspects potentially leading to a significantly underestimated evaluation, as compared to the potentially real one, namely:

- In 2.4% of cases, operators who assessed the images asserted the importance of highlighting defects potentially leading to data misinterpretation. However, it is impossible to determine the number of examiners who did not identify potential defects.
- Reporting of defects may have been omitted by experienced operators relying on their personal expertise.
- Reporting of defects may have been omitted by less experienced operators who were unable to recognize the problem.
- Although 2.4% may appear to be a small percentage, it is important to emphasize that this percentage, within a sample of over 1,200,000 cases, translates to approximately 30,000 potential misdiagnoses, with all the associated consequences.
- If only Positron Emission Tomography (PET), CT, and Magnetic Resonance Imaging (MRI) are considered, the percentage of defects potentially leading to data misinterpretation highlighted by the operators increases up to 6%. This means that these last techniques, typically adopted for automatic cancer detection, are more prone to being affected by alterations during acquisition.

Anyway, radiologists typically attempt to analyze images and provide a diagnosis, even in cases of low-quality images, frequently documenting the details concerning poor quality directly in the report [37].

The prevalence of concerns about image quality (Figure 3c), as reported in clinical evaluations, underscores the critical need for robust assessment methods to ensure the reliability of radiomic data. In cases where artifacts in images are not identified, failure to address the above issues can lead to misinterpretation of radiomic features and subsequent misdiagnosis or delayed treatments [38]. Furthermore, the inherent subjectivity in detecting image artifacts highlights the importance of objective assessment tools to standardize quality evaluation across different radiology practices and expertise levels. In addition to technical aspects, there is also an economic need. Even if investing resources in high-quality imaging technologies for radiomics involves upfront costs related to purchasing equipment, training personnel, and potentially increased operational costs, these investments can lead to significant economic benefits in the long term. By improving diagnostic accuracy and efficiency, high-quality imaging can reduce unnecessary procedures, misdiagnoses, and treatment complications. It can also enable personalized treatment plans that are more cost-effective and have better outcomes, potentially leading to overall healthcare cost savings.

## 6. Main Concerns in Clinical Practice

An artifact in imaging refers to any distortion or anomaly present in medical images, leading to non-accurate representation of the underlying anatomy or pathology of the scanned tissue or organ [39]. These discrepancies can emerge from different sources, including the imaging process itself, and other patient-related factors. Artifacts may reveal unexpected shadows, streaks, blurs, or other forms of distortion, that potentially could lead to misleading diagnoses or treatment planning. Table 1 summarizes the most common types of imaging artifacts, categorizing them by imaging modality. Their presence is a significant concern in radiology and medical imaging analysis, with worrying rates in some settings, such as the reported 38% of cases in [40].

### 6.1. Motion

**Figure 4:**
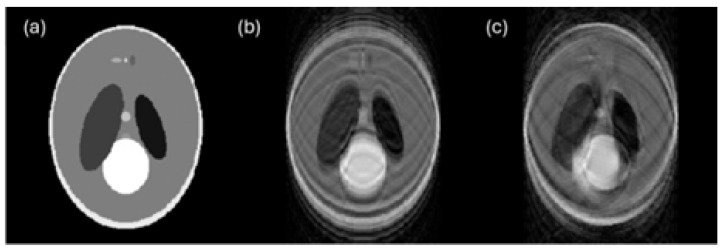
Motion artifacts: (**a**) original image; (**b**) motion artifact due to translational motion; and (**c**) motion artifact due to rotational motion [47].

In medical imaging, particularly with PET/CT and MRI modalities, patient motion is the main source of artifacts that significantly degrade image quality (Figure 4) [41,42]. The long acquisition times required for these modalities amplify the issue, with MRI being notably sensitive due to its complex nature and the extended duration needed to capture high-quality images [43]. This problem is not confined to a specific patient demographic; however, it is pronounced in elderly patients with movement disorders [41] and highly kinetic participants such as those in developing and aging populations. As reported in [43], various techniques have been proposed to address motion artifacts, including mechanical restraints, synchronized sensors, and advanced computational methods, such as machine learning and image processing. Additionally, the advent of simultaneous PET-MR imaging offers new ways for motion correction, exploiting MR-based techniques for real-time monitoring and correction during PET data acquisition [44]. Despite these advancements, motion correction in medical imaging remains a challenging field, with no single method proving to be effective in all situations, underscoring the need for continuous research and development in this area [45,46].

### 6.2. Beam Hardening Streaks and Cupping Artifacts

**Figure 5:**
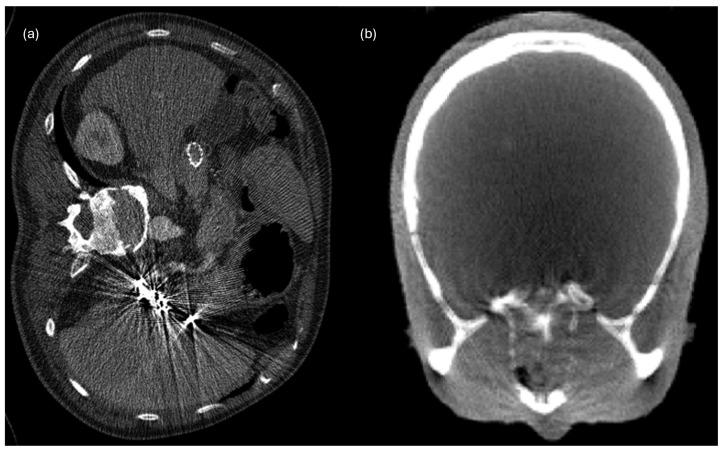
Imaging artifacts: (**a**) streak artifact due to embolization coils [49]; and (**b**) axial view of a skull phantom affected by cupping artifacts [50].

Beam hardening artifacts, specifically in CT scans, are substantial due to the polychromatic nature of X-ray beams. As these beams penetrate the body, lower-energy photons are absorbed more readily than higher-energy photons, causing the beam to harden as it passes through the tissue [48]. This hardening alters the expected attenuation profile, producing streak and cupping artifacts, which can lead to significant image distortions and can affect clinical diagnoses and quantitative radiomic analyses [27]. Streaking artifacts appear as lines or streaks in the CT image, and they are especially noticeable around high-density objects within the scan, such as metal implants (Figure 5a [49]). The cupping effect is observed as an abnormal, cup-shaped distortion in the homogeneity of an image, particularly in the case of homogeneous substances such as soft tissues (Figure 5b [50]). Various approaches exist to mitigate the above artifacts. Traditionally, hardware-based solutions such as iterative reconstruction techniques and dual-energy CT have been employed [51,52]. These methods adjust the CT data acquisition process, or they use computational techniques to compensate for the artifacts; however, they often involve significant computational resources or additional hardware, which may not always be available in clinical settings [53]. Alternatively, recent advancements have introduced software-based solutions using artificial intelligence (AI) to address the problem of beam hardening. These AI models, such as the one proposed by Kalare et al. [27], take advantage of large datasets of CT images to learn the characteristics of beam-hardening artifacts and apply corrections during the image reconstruction process. The AI-driven approach is designed to improve the accuracy of the reconstructed images, thereby enhancing the quality of radiomic data for better clinical and research outcomes [54].

### 6.3. Noise

**Figure 6:**
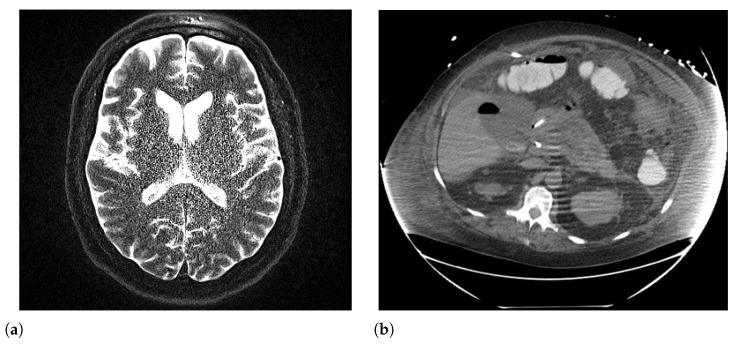
(**a**) MRI of the human brain corrupted by noise [63]; (**b**) streak artifacts generated by large body habitus [64].

Noise in radiomics refers to random variations in the gray level of image data pixels, which are not related to the true signal (Figure 6). Noise in radiomic data can originate at different stages of the imaging pipeline, each characterized by distinct physical or algorithmic mechanisms, and by a specific impact on the stability of quantitative features. In particular, it is possible to distinguish between acquisition noise, reconstruction noise, and post-processing noise. Acquisition noise is intrinsic to the image formation process, and it is primarily determined by the physical limitations of the detection system. This type of noise shows a direct dependence on parameters such as radiation dose, acquisition time, or signal-to-noise ratio, and it tends to manifest as an increase in voxel-wise variability [55]. The noise introduced during the reconstruction phase, in addition to representing the propagation of acquisition noise, is conditioned by algorithmic choices. Iterative reconstruction methods, as well as the use of different reconstruction kernels or smoothing levels, can modify the spatial distribution of noise, introducing medium-range correlations or structured artifacts [56]. The reproducibility of radiomic features in noisy images is commonly assessed using techniques such as test-retest studies, phantom studies, and statistical stability analysis. These methods evaluate whether features extracted from the same region of interest remain consistent under different conditions or across multiple imaging sessions [12]. Studies have identified several radiomic features that are robust to noise, including shape-based features and some textural features, such as entropy and energy. These features tend to exhibit less variation under different noise levels compared to more complex textural features, such as local binary patterns or high-order statistical features [57]. Generative models, particularly those based on deep learning, show promise in improving the quality of low-dose CT images by reducing noise and enhancing relevant structures. These models, such as Generative Adversarial Networks (GANs) and Variational Autoencoders (VAEs), learn to generate or reconstruct images that are close to high-dose or high-quality images, thus preserving important radiomic features while reducing noise [24]. The aforementioned generative models demonstrate promising capabilities for image denoising and quality enhancement, but their application in radiomics workflows raises important methodological concerns that should be carefully considered. A critical risk associated with deep learning-based correction methods is the potential introduction of feature hallucination or distribution shift, i.e., alterations to texture, intensity patterns, or spatial correlations not perceptually obvious but significantly affecting extracted radiomic features [58,59]. In particular, GANs are trained to generate visually “plausible” images by learning statistical patterns from training data. However, this process can introduce synthetic texture components or may suppress tissue heterogeneity in such a way as to alter radiomic features such as Gray-Level Co-occurrence Matrices (GLCM), Gray-Level Run-Length Matrices (GLRLM), and wavelet-based descriptors [11,58]. As an example, GAN-based denoising may inadvertently smooth tumor heterogeneity patterns that are clinically relevant for prognosis prediction, or conversely, they can introduce artificial texture patterns not present in the original anatomy [59,60]. These alterations can propagate through the entire radiomics pipeline, potentially leading to overfitted models, loss of generalizability, or biased clinical predictions when deployed on uncorrected images. In contrast, traditional correction methods, such as wavelet-based denoising [61], or iterative reconstruction algorithms [62], operate on solid mathematical principles with predictable effects on image statistics. These approaches may be less effective compared to deep learning methods when noise is severe, but they typically preserve feature distributions more consistently, and they have a reduced risk of unpredictable feature alterations. However, traditional methods also have limitations: they may require manual tuning, exhibit reduced effectiveness, or introduce artifact patterns.

### 6.4. Blur

Image blur significantly impacts the accuracy of texture analysis in radiomic studies, as it can obscure or alter image details and texture, similar to other artifacts. Common sources of blur include patient movement, system resolution, imaging technique variations, and processing algorithms. Consequently, blur can lead to increased false positive or false negative rates in classifying lesions [65]. Without addressing blur, the extracted features may not accurately reflect the true pathological condition, potentially leading to incorrect disease staging, incorrect treatment planning, and poor patient outcomes. Effective methods for correcting blur in radiomic feature extraction include deconvolution techniques [66] and the use of sharpness enhancement algorithms [67]. From a radiomics perspective, image blur reduces high-frequency spatial information, introducing systematic bias and reduced stability, in particular for texture-based and higher-order radiomic features, and it may also affect shape features through inaccurate lesion boundary delineation [66].

### 6.5. Body Habitus/Patient Size

Overweight and obese patients increasingly present imaging challenges that can degrade image quality in diagnostic radiology (Figure 6b) [64,68], leading to increased image noise, reduced contrast, beam hardening artifacts, and streak artifacts, which can obscure anatomical structures and compromise diagnostic accuracy [69,70,71]. As patient thickness increases, photon attenuation also rises exponentially. Therefore, the goal in imaging obese patients should be to enhance image quality in accordance with the ALARA (As Low As Reasonably Achievable) principle [72]. These issues can complicate the interpretation of images in routine clinical practice, thus affecting the accuracy of feature extraction used to predict tumor behavior [73]. Additionally, there is a recognized link between diagnostic radiation from CT scans and a small, yet heightened, risk of cancer, particularly among younger populations [74]. Beyond the direct impact on image noise and artifact generation, patient size and body habitus introduce an additional level of complexity in radiomics analyses because of their interaction with scanner exposure control systems and reconstruction parameters. Scanners that employ Automatic Tube Current Modulation (ATCM) or Automatic Exposure Control (AEC) to dynamically adjust the radiation dose based on patient attenuation have demonstrated that AEC efficiency is strongly dependent on patient body size, with potential dose increases in certain patient populations [75].

## 7. The Impact of Image Quality on Segmentation

Image segmentation is a computer vision technique that aims to divide a digital image into distinct groups of pixels with the same characteristics, known as segments. This technique is crucial for object detection and similar tasks because it simplifies the image’s complex visual information into more manageable sections. Through image segmentation, the process of image analysis becomes quicker and more sophisticated, thus facilitating advanced image processing techniques. Factors such as noise, contrast, and artifacts have a direct impact on the performance of segmentation algorithms [76]. The segmentation accuracy also largely depends on the algorithm’s ability to identify and delineate objects, which is influenced by these image quality factors. Therefore, enhancing image quality through methods such as noise reduction and contrast enhancement can directly improve segmentation outcomes, underscoring image quality as a critical determinant of the success of image segmentation [77,78]. In diagnostic radiology, where images are analyzed to support decision-making in clinical practice, precision in segmenting anatomical structures from medical images is crucial for accurate delineation, disease diagnosis, treatment planning, and monitoring, ensuring effective patient care [79]. The accuracy of segmentation directly influences the reliability of subsequent radiomic feature extraction and, by extension, the utility of these features as biomarkers for disease characterization, prognosis, and prediction of therapy response [80]. It should be noted that different segmentation strategies such as manual delineation, semi-automatic methods, and fully automatic deep learning-based approaches exhibit sensitivity that varies with image quality degradations. Manual segmentation, while time-consuming, may benefit from expert visual compensation for artifacts; radiologists can exploit anatomical knowledge to interpret degraded regions, although image alterations may still introduce diagnostic uncertainty. Fully automatic deep learning-based segmentation models, while offering speed and consistency, can be highly sensitive to deviations in image quality characteristics that differ from their training distributions. This sensitivity can lead to segmentation failures or systematic biases when deployed on data acquired with different protocols or scanner settings. Consequently, the interaction between image quality and segmentation accuracy introduces an additional source of uncertainty in radiomic feature extraction, underscoring the critical importance of robust IQA protocols applied upstream of the segmentation step [11,76,81]. Image quality plays a pivotal role in guaranteeing the precision of image segmentation [82]. Moreover, image artifacts can lead to the extraction of unreliable radiomic features, as these features are highly sensitive to the quality and dimensions of the segmented region [83,84,85]. Features such as texture, shape, and intensity distribution are directly affected by segmentation quality, and any errors in segmentation can propagate through to the extracted features, driving misleading conclusions about the presence or characteristics of disease (Table 2).

## 8. Image Quality Improves Radiomic Biomarker Extraction

Image quality is critical for the fidelity of radiomics biomarkers, emphasizing the importance of optimizing imaging protocols to maximize information content while minimizing artifacts and distortions. High-quality imaging data enable the extraction of subtle and clinically relevant features, facilitating more accurate tumor characterization and therapy planning in oncology. Furthermore, standardization initiatives such as the Image Biomarker Standardization Initiative (IBSI) [18] emphasize the crucial role of image quality in ensuring consistency and reproducibility across different imaging platforms and clinical settings [22]. In addition, IBSI Phase 2 extends these standardization efforts to convolutional image filters commonly used in radiomics pipelines, such as wavelet and Laplacian-of-Gaussian filters, providing reference filtered images and reference feature values to improve the reproducibility of filtered radiomic features [22]. Cobo et al. [86] also emphasized that variations in image quality can significantly affect radiomic feature stability and reproducibility, highlighting the importance of standardized imaging protocols to maintain consistency in radiomic analyses [23,87,88]. The implementation of standardized reporting frameworks has become essential for ensuring methodological rigor in radiomics research. The Transparent Reporting of a multivariable prediction model for Individual prognosis Or Diagnosis (TRIPOD) statement provides comprehensive guidelines for reporting predictive models [89], while the Checklist for Artificial Intelligence in Medical Imaging (CLAIM) framework specifically addresses AI-based imaging studies [90]. Finally, in the context of radiomics research, the CheckList for EvaluAtion of Radiomics research (CLEAR) is designed to assess the repeatability and reproducibility of radiomic features, providing detailed guidance on the key stages of the radiomics pipeline, which include image acquisition, segmentation, preprocessing, feature extraction, and statistical analysis [23]. These frameworks complement IBSI standardization by ensuring transparent and reproducible research practices. By establishing standardized imaging protocols and quality control measures, it will be possible to mitigate variability introduced by imaging artifacts and scanner-dependent factors, thereby enhancing robustness and generalization of radiomics biomarkers. Moreover, Larue et al. [91] highlight the impact of image quality parameters, such as gray level discretization, on the stability and reliability of radiomics features. Similarly, Yadav et al. [92] conducted a phantom study to systematically evaluate how acquisition parameters such as slice thickness, dose, and reconstruction algorithm affect radiomic feature stability, identifying metrics robust to such variations. Their comprehensive phantom study underscores the importance of optimizing imaging parameters to minimize variability and maximize feature stability, thus improving the clinical utility of radiomics biomarkers [93]. Improved image quality enhances the delineation of anatomical structures and pathological regions, thus enabling more precise feature extraction. Furthermore, suitable image quality minimizes artifacts and distortions, ensuring the fidelity of radiomic features and reducing variability in quantitative measurements. While most conventional IQA metrics are designed to capture perceptual or global image degradations, several emerging approaches have been proposed to assess image quality directly in terms of radiomic feature robustness. In particular, perturbation-based stability analyses have been increasingly adopted as a practical surrogate for test-retest experiments, enabling the evaluation of feature sensitivity to controlled variations in noise, spatial resolution, voxel resampling, and segmentation uncertainty [18]. By computing stability metrics, such as the Intraclass Correlation Coefficient (ICC) across perturbed images, these methods allow the identification of features that remain robust under clinically relevant quality variations. Complementary to perturbation strategies, simulation and phantom-based frameworks have been proposed to systematically characterize radiomic feature sensitivity to acquisition and reconstruction parameters, under controlled conditions [94,95]. In this context, the Intraclass Correlation Coefficient (ICC) [96] is widely employed in radiomics studies as a practical strategy to assess feature robustness and repeatability, particularly in test-retest, multi-observer, and multi-scanner settings. Across the literature, ICC-based analyses are commonly used to exclude unstable or noise-sensitive features, with reported thresholds typically ranging between 0.7 and 0.9. However, several methodological works emphasize that ICC alone may be insufficient to fully characterize feature robustness, as it depends on the underlying data variance and acquisition conditions, and should therefore be interpreted in conjunction with image quality considerations and complementary standardization frameworks [11,12,13,14]. From an operational perspective, Image Quality Assessment should therefore be considered as a preliminary quality-control and gating step within the radiomics pipeline, to be applied before segmentation and feature extraction to identify unsuitable images for quantitative analysis, as well as to contextualize feature stability analyses, rather than as a direct modeling variable. In conclusion, the evidence from these studies collectively supports the notion that image quality improvements play a critical role in enhancing the extraction and utility of radiomics biomarkers, contributing to improved patient stratification, treatment response assessment, and personalized medicine in clinical practice [97]. A summary of the main image quality degradation sources and the corresponding mitigation strategies adopted in radiomics workflows is reported in Table 3.

## 9. Image Quality Metrics

Considering the significant impact of image quality on radiomic analyses, and the dependency of extracted information reliability on such quality, establishing metrics to accurately evaluate an image’s usability is fundamental [98]. Therefore, the development, selection, and implementation of appropriate metrics become critical to ensure data consistency, comparability, and ultimately, diagnostic reliability [99]. Given the diverse imaging conditions and acquisition protocols in clinical practice, robust metrics are required to assess whether an image is suitable for radiomic analysis. Traditional metrics such as Signal-to-Noise Ratio (SNR), Spatial Resolution, Contrast-to-Noise Ratio (CNR), and Uniformity have been widely adopted [100,101,102]. More recently, advanced metrics like the Learned Perceptual Image Patch Similarity (LPIPS) [103] and no-reference quality estimators like Blind/Reference less Image Spatial Quality Evaluator (BRISQUE) [104,105] have demonstrated good performance in detecting image degradations. Radiomic-specific IQA metrics should be designed to reflect not only perceptual image fidelity, but also their impact on downstream radiomic analyses. Conventional IQA measures, largely developed for visual assessment or general image processing tasks, may fail to capture degradations that minimally affect human interpretation yet substantially alter radiomic feature values, segmentation accuracy, or model robustness. For this reason, the clinical and methodological relevance of radiomic-specific IQA metrics should be evaluated through their association with quantitative endpoints intrinsic to the radiomics pipeline. Potential validation strategies include assessing the relationship between IQA scores and radiomic feature reproducibility (e.g., intraclass correlation coefficients), analyzing the sensitivity of segmentation performance metrics (e.g., Dice similarity coefficient) to controlled variations in image quality, and comparing feature stability before and after quality-improving interventions, such as denoising or harmonization. Graphical analyses, such as feature stability curves or correlation plots between IQA scores and downstream performance metrics, may provide an intuitive and reproducible framework for testing the effectiveness of radiomic-oriented IQA measures. Such approaches would allow future metrics to be benchmarked not only against visual quality, but against their ability to preserve the reliability and clinical interpretability of radiomic biomarkers.

### Full-Reference vs. No-Reference Metrics

Image quality metrics can be classified into two main categories: full-reference (FR) and no-reference (NR) metrics. Full-reference metrics require a “pristine” reference image for comparing and quantifying the deviation of the target image from this ideal. These include metrics such as Peak Signal-to-Noise Ratio (PSNR), Structural Similarity Index (SSIM) [106], and Learned Perceptual Image Patch Similarity (LPIPS) [103]. However, their application in radiomics is limited due to their full-reference nature. A critical question in IQA is what can be considered a true “reference” image when using FR metrics; the definition of such a reference in clinical practice remains ambiguous and context-dependent. In practical workflows, the reference is implicitly defined by expert clinical judgment, where a radiologist evaluates an image as being of “diagnostically acceptable” quality. In this context, the gold standard becomes human interpretation, which introduces subjectivity and inter-observer variability.

NR metrics, on the other hand, evaluate image quality directly from an image without any ground truth comparison. These are significantly more practical in medical imaging pipelines, particularly for large-scale clinical datasets, where reference images are rarely available. Traditional metrics such as SNR, CNR, Spatial Resolution, and Uniformity fall within this category and are frequently used in radiomics literature [100,101,102]. Among the NR metrics, BRISQUE has gained attention in image processing. BRISQUE assesses image quality by extracting natural scene statistics (NSS) from image patches and comparing them to statistical models of distortion-free images. It has been shown to effectively capture blur, noise, compression artifacts, and contrast loss without the need for a reference image. While BRISQUE offers the clear advantage of being a no-reference metric, it also presents certain limitations. Specifically, BRISQUE provides an overall image quality score without offering detailed information about the nature or source of the degradation. This lack of interpretability makes it less effective in scenarios where the identification of specific artifacts is needed. It is essential to clarify that the IQA metrics discussed in the present review (SNR, CNR, SSIM, BRISQUE, LPIPS) are designed to assess image-level quality degradations rather than to directly quantify the stability or reproducibility of radiomic features themselves. These metrics serve as upstream quality control filters to identify images with fundamental acquisition issues that would preclude reliable downstream analysis. However, traditional metrics present critical limitations when applied to radiomics workflows. SNR and CNR provide global intensity assessments, but they both fail to capture spatially localized texture disruptions that disproportionately affect second-order radiomic features such as GLCM and GLRLM [11]. The analysis of PET radiomics studies revealed that spatial resolution has the strongest effect on radiomic feature variability, underscoring that local spatial information is more critical for texture features than global intensity metrics suggest [58]. SSIM, while effective for perceptual quality tasks, exhibits decreased correlation when applied to blurred and noisy images and performs poorly for recognition threshold tasks [106]. BRISQUE, though suitable for no-reference assessment, requires retraining for medical images, since it was originally designed for natural scene statistics [105]. The fundamental limitation is that passing traditional IQA thresholds represents a necessary but not sufficient condition for robust radiomics. An image may achieve acceptable SNR or BRISQUE scores yet still yield unstable texture features due to inconsistent processing conditions such as quantization range, bin number, and reconstruction parameters, which directly affect probability distributions underlying second-order feature calculations [91].

## 10. Conclusions

This work provides a critical overview of measurement methods for assessing image quality in radiomics. While previous systematic reviews have addressed radiomic feature reproducibility or harmonization strategies, this review systematically quantifies the prevalence of IQA consideration in radiomics literature from a metrological standpoint. Analysis of the recent literature indicates that research efforts to date have largely focused on the development of automated tools for disease detection and evaluation. Very few studies address the correlation between poor-quality images and their effect on disease detection, both in human interpretation and automated analysis. The reviewed literature reports that approximately 2.4% of 1,210,000 radiology images were affected by alterations, corresponding to approximately 30,000 cases in which image quality could contribute to misinterpretation and potential misdiagnosis. It is worth noting that this percentage derives from a heterogeneous clinical cohort that spans multiple imaging modalities and clinical contexts. Direct extrapolation of these figures should be carefully interpreted, as the prevalence and impact of quality issues may vary depending on imaging protocols, scanner technologies, clinical applications, and the specific adopted feature extraction pipelines. Nevertheless, this underscores the substantial clinical and economic burden of undetected or unaddressed image quality degradation in quantitative imaging analyses. Moreover, it must be highlighted that this number may substantially increase in the case of PET, CT, and MRI, which are widely used in tumor detection. When the analysis is performed by human operators, the clinical results include notes about poor image quality, strongly affecting the reliability of the diagnosis. This overview has also reported the main alterations by classifying them according to the radiomic image type they affect. The most commonly used metrics for assessing image quality are reported as well. Despite the critical role of these metrics, there are notable gaps and challenges when applying them to radiomics [107]. Many existing metrics were developed for visual IQA, not for quantitative analysis of high-dimensional data extracted from images [91]. Another challenge is the variability in imaging conditions and protocols across different institutions and even within the same institution over time. This variability can lead to significant differences in image quality and, consequently, in the extracted radiomic features, thus complicating multicenter studies and the generalization of radiomic models [28,29,30]. Moreover, the interaction between image quality metrics and radiomic features is complex and not fully understood [108]. Some radiomic features may be more sensitive to changes in certain image quality metrics than others, and the impact of this sensitivity on clinical decision-making is still being investigated [109]. It should be acknowledged that radiomic feature reliability is strongly modality-dependent, introducing an additional layer of complexity in interpreting aggregated quality metrics across heterogeneous imaging studies. Cross-sectional imaging modalities such as CT and MRI, which rely on standardized acquisition protocols and well-controlled physical parameters, generally demonstrate higher feature stability and reproducibility compared to operator-dependent or real-time imaging techniques such as ultrasound. Future work should take into account radiomic feature reproducibility across modalities using standardized phantoms and harmonized protocols, as recommended by initiatives such as IBSI, to establish specific quality thresholds and feature selection criteria in different modalities. Future systematic reviews could complement this work by performing stratified quantitative analyses that link specific artifact types to feature-class stability (e.g., first-order, texture, higher-order features) across imaging modalities, using standardized phantom datasets and harmonized feature extraction protocols. Due to the metrological nature of the review, meta-analysis and formal risk of bias assessment are omitted, but they will be considered in a future applied paper. To address these gaps, there is a growing demand for the development of new metrics and standards that are specifically designed for radiomics [107,110,111]. Addressing this need will be crucial for unlocking the full potential of radiomics in clinical applications, ensuring that the quantitative features extracted from medical images are accurate, reliable, and meaningful for patient care.

## Figures and Tables

**Table 1:** Typical concerns arising during image acquisition and formation, categorized by imaging modality. A check mark indicates that the modality is typically affected by the artifact.

Concern	CT	PET	MRI	X-Rays	Ultrasound	Typical Source
Motion	✓	✓	✓	✓	✓	Patient movement, long acquisition times
Beam hardening	✓	✓		✓		Polychromatic X-ray beams, high-density materials
Noise	✓	✓	✓	✓	✓	Low dose, acquisition settings, patient size
Blur	✓	✓	✓	✓	✓	Motion, system resolution limits, reconstruction effects
Patient size	✓	✓		✓	✓	Photon attenuation, scattering, reduced signal-to-noise ratio

**Table 2:** Radiomic feature classes affected by image artifacts.

Artifact Type	Imaging Modality	Affected Feature	Reported Impact
Motion	MRI, PET, CT	Texture, shape	Reduced reproducibility, segmentation errors
Beam hardening	CT	First-order, texture	Feature bias, intensity distortion
Noise	CT, MRI, PET	Texture (GLCM, GLSZM), first-order	Increased variance, instability
Blur	CT, MRI, PET	Texture, shape	Loss of edge definition, misclassification
Body habitus	CT, PET	First-order, texture	Contrast loss, increased noise

**Table 3:** Mitigation strategies for image artifacts in radiomics.

Artifact Type	Mitigation Approach	Level of Application	Limitations
Motion	Motion correction, AI-based methods	Image-level	Computational cost, modality dependence
Beam hardening	Dual-energy CT, iterative reconstruction, deep learning	Acquisition/reconstruction	Hardware availability, training data
Noise	Filtering, GAN-based denoising	Image-level	Possible alteration of texture features
Blur	Deconvolution, sharpness enhancement	Image-level	Requires PSF estimation
Scanner/protocol variability	Statistical harmonization	Feature-level	Requires batch definition, possible signal masking

## Data Availability

No new data were created or analyzed in this study. Data sharing is not applicable to this article.

## Abbreviations

The following abbreviations are used in this manuscript:

IQA	Image Quality Assessment
CT	Computed Tomography
MRI	Magnetic Resonance Imaging
PET	Positron Emission Tomography
AI	Artificial Intelligence
GAN	Generative Adversarial Network
VAE	Variational Autoencoder
SNR	Signal-to-Noise Ratio
CNR	Contrast-to-Noise Ratio
SSIM	Structural Similarity Index
LPIPS	Learned Perceptual Image Patch Similarity
BRISQUE	Blind/Referenceless Image Spatial Quality Evaluator
IBSI	Image Biomarker Standardization Initiative
TRIPOD	Transparent Reporting of a Multivariable Prediction Model for
	Individual Prognosis or Diagnosis
CLAIM	Checklist for Artificial Intelligence in Medical Imaging
ALARA	As Low As Reasonably Achievable
FR	Full-Reference
NR	No-Reference
ICC	Intraclass Correlation Coefficient
GLCM	Gray-Level Co-occurrence Matrix
GLRLM	Gray-Level Run-Length Matrix
PRISMA	Preferred Reporting Items for Systematic Reviews and Meta-Analyses
QUADAS-2	Quality Assessment of Diagnostic Accuracy Studies, 2nd edition
GRADE	Grading of Recommendations Assessment, Development and Evaluation
